# A Systematic Review and Meta-analysis of the Efficacy and Safety of Lytic and Non-lytic Early Thrombus Removal Technologies for Iliofemoral Deep Vein Thrombosis

**DOI:** 10.1097/SLA.0000000000006765

**Published:** 2025-05-27

**Authors:** Benedict R.H. Turner, Sara Jasionowska, Jessica Shea, Avik Ghosh, Matthew Machin, Adam M. Gwozdz, Stephen A. Black, Alun H. Davies

**Affiliations:** * Section of Vascular Surgery, Department of Surgery and Cancer, Imperial College London, London, UK; † Department of Orthopaedic Surgery, Kingston Hospitals NHS Foundation Trust, Kingston Upon Thames, UK; ‡ Academic Department of Vascular Surgery, St Thomas’s Hospital, King’s College London, London, UK

**Keywords:** anticoagulation, catheter directed thrombolysis, deep vein thrombosis, iliofemoral, mechanical thrombectomy, pharmacomechanical thrombectomy, post-thrombotic syndrome, pulmonary embolism, venous thromboembolism

## Abstract

**Objectives:**

This systematic review and meta-analysis compared the effectiveness and safety of lytic and non-lytic early thrombus removal strategies in addition to anticoagulation versus anticoagulation alone.

**Background:**

Early thrombus removal strategies have been developed to prevent post-thrombotic syndrome (PTS) following acute iliofemoral deep vein thrombosis (DVT).

**Methods:**

This review followed PRISMA guidelines using a registered protocol (CRD42023437158). The MEDLINE and Embase databases, as well as trial registries, were searched without limitations. Head-to-head or single-armed trials or studies that reported the rate of PTS in patients with iliofemoral DVT (symptomatic for <28 days) and early thrombus removal were included. The rates of PTS, moderate-severe PTS, major bleeding, risk-benefit ratio, DVT recurrence, and mortality were pooled in meta-analysis with fixed or random effects.

**Results:**

Across all study designs (20 studies), the rate of PTS was 24.5% (95% CI: 19.5–30.3%) for lytic therapies, 18.8% (1 study) for non-lytic therapy, and 40.4% (95% CI: 35.3–45.7) for anticoagulation alone. The number needed to treat was 6 for PTS and 15 for moderate-severe PTS. In randomized trials, the odds of major bleeding with lytic therapies were 4.9 (95% CI: 1.3–19.1) compared with anticoagulation; the number needed to harm was 33. There was no major bleeding for mechanical thrombectomy.

**Conclusions:**

Early thrombus removal reduces PTS and moderate-severe PTS, while increasing nonfatal major bleeding. Mechanical thrombectomy removes major bleeding risk, but efficacy evidence is limited to 1 observational study.

Venous thromboembolism (VTE) is the third most common cardiovascular disease in both incidence and mortality.^1^ Early thrombus removal technologies have been developed, particularly focusing on the treatment of deep vein thrombosis (DVT), and a number of randomized controlled trials (RCTs) have assessed the efficacy and safety of these treatments.^2–5^ DVT is the most common manifestation of VTE, with a lifetime incidence of 2% to 5% and high morbidity in the form of the post-thrombotic syndrome (PTS).^6^ This common, chronic, and disabling complication of DVT occurs in 20% to 50% of patients, depending on anatomic location and is associated with leg pain, swelling, venous claudication, and, in severe cases, progression to venous ulceration.^7,8^ Particularly, thrombosis of the external/common iliac vein with adjacent femoral thrombosis, so-called iliofemoral DVT (IF-DVT), is less likely to regress spontaneously due to a failure of recanalization in 60% to 80% and is more strongly linked to PTS with more severe PTS symptoms.^8–12^ PTS has severe and profound effects on quality of life, akin to heart failure or cancer, with a considerable economic impact thereafter.^8,13^

Early thrombus removal technologies aim to prevent the development of PTS, as per the Open Vein Hypothesis.^14^ Specific technologies that may be utilized include catheter-directed thrombolysis (CDT), pharmacomechanical catheter-directed thrombolysis, and ultrasound-accelerated catheter-directed thrombolysis, which hereafter are referred to collectively as lytic therapies, and more recently, purely mechanical thrombectomy (non-lytic). Though some evidence for efficacy has previously been demonstrated, the significant risk of major bleeding has prompted significant heterogeneity in the recommendations for lytic-based therapies in international guidelines.

NICE guidelines on mechanical thrombectomy state that special arrangements for clinical governance, consent, and audit or research are currently required and recommend lytic therapies for people with symptomatic acute IF-DVT (<14 days), who have good functional status and a low risk of bleeding.^15^ The American College of Chest Physicians guidelines recommend anticoagulation alone over lytic therapies due to the risk of major bleeding.^16^ The European Society for Vascular Surgery (ESVS) guideline on venous thrombosis concluded that there was good quality evidence that catheter-based interventions prevented moderate-severe PTS, with a modest increase in bleeding risk from a meta-analysis of 4 RCTs for proximal DVT, which included both iliofemoral and femoropopliteal subgroups.^17^ IF-DVT patients carry the greatest risk of PTS compared with femoropopliteal DVT, and the recent increase in the use of purely mechanical thrombectomy, which extends the treatment window for lysis for acute IF-DVT, has warranted reviewing this subject area. Therefore, a systematic review and meta-analysis of all studies of early thrombus removal for IF-DVT was conducted to understand the effectiveness and safety of current early thrombus removal treatments and to inform both patients and clinicians of the risks and benefits.

## METHODS

This review was conducted according to the Preferred Reporting Items for Systematic Reviews and Meta-analyses (PRISMA, Supplemental Digital Content 1, http://links.lww.com/SLA/F493) guidelines with a registered protocol (PROSPERO: CRD42023437158).^18^

### Search Strategy

Online databases (MEDLINE, EMBASE, and the Cochrane Library) were searched up to February 14, 2024. Searches were carried out without any restrictions regarding publication type or language. Gray literature was screened by searching unpublished trials on ClinicalTrials.gov, European Union Clinical Trials, and the International Standard Randomised Controlled Trial Number registry. Medical subject heading (MESH) terms and free word searches were used; the full search strategy is available in Supplementary Table 1, Supplemental Digital Content 2, http://links.lww.com/SLA/F494. References of the included articles were screened for further eligible studies. Two authors (S.J. and B.R.H.T.) independently screened the identified articles against the inclusion criteria, and any disputes were mediated by a third author (A.M.G.). The screening process was conducted on the EndNote X20 (Clarivate); titles and abstracts were screened before full-text review.

### Eligibility Criteria

The included articles had the following characteristics:

1. early thrombus removal strategy;
2. diagnosis, presentation, and treatment within 28 days;
3. reporting the rate of PTS with at least 6 months follow-up; and
4. any study design.

Excluded articles had the following characteristics:

1. no follow-up;
2. DVT of symptom duration >28 days;
3. non–IF-DVT;
4. <75 patients; and
5. conference abstracts, duplicate publications, or articles in which the full text is not available in English.

### Data Extraction and Analysis

Data from the included articles were extracted using a standardized template in Microsoft Excel by 2 authors (S.J. and B.R.H.T.) independently. The International Society on Thrombosis and Hemostasis (ISTH) definition of major bleeding was used to standardize the reporting of bleeding events.^19^ Where data for the IF-DVT subgroup were not immediately available, study authors were contacted up to 3 times to invite submission of data for inclusion in the review. Statistical analyses were conducted using R version 3.3.2 (R Core Team, GNU GPL v2 License), R Studio version 1.0.44 (RStudio Inc., GNU Affero General Public License v3, 2016), and the *meta* package. Where data from the 2 study arms were available, a comparative head-to-head meta-analysis was conducted with fixed effects. The indicators were reported as odds ratios. For single-arm studies, frequency estimates were pooled and reported as event rates in meta-proportions analysis with associated 95% CIs. Statistical heterogeneity was assessed using the *I* ^2^ test, and meta-regression was performed to account for heterogeneity in results. A random-effects meta-analysis was conducted; where event proportions for individual studies were low, a logit transformation was used to normalize the data.

### Outcomes

The primary efficacy outcome was the rate of PTS, and the primary safety outcome was the rate of major bleeding. Secondary outcomes included the rate of moderate-severe PTS, risk-benefit ratio, the rate of ulceration, the rate of peri-procedural recurrence and later DVT recurrence, treatment-related mortality, venous patency at follow-up, and a meta-regression analysis of how follow-up duration affects the rate of PTS development. Where multiple studies of the same patient cohorts were published, the study with the longest possible follow-up was used.

### Quality and Risk of Bias Assessment

The Cochrane Risk of Bias tool was used to evaluate RCTs, and the ROBINS-I tool was used for nonrandomized studies. The Grading of Recommendations, Assessment, Development and Evaluations Framework (GRADE) was used to assess the quality and certainty of the evidence, using an online platform, GradePro (Evidence Prime Inc.).

## RESULTS

**FIGURE 1:**
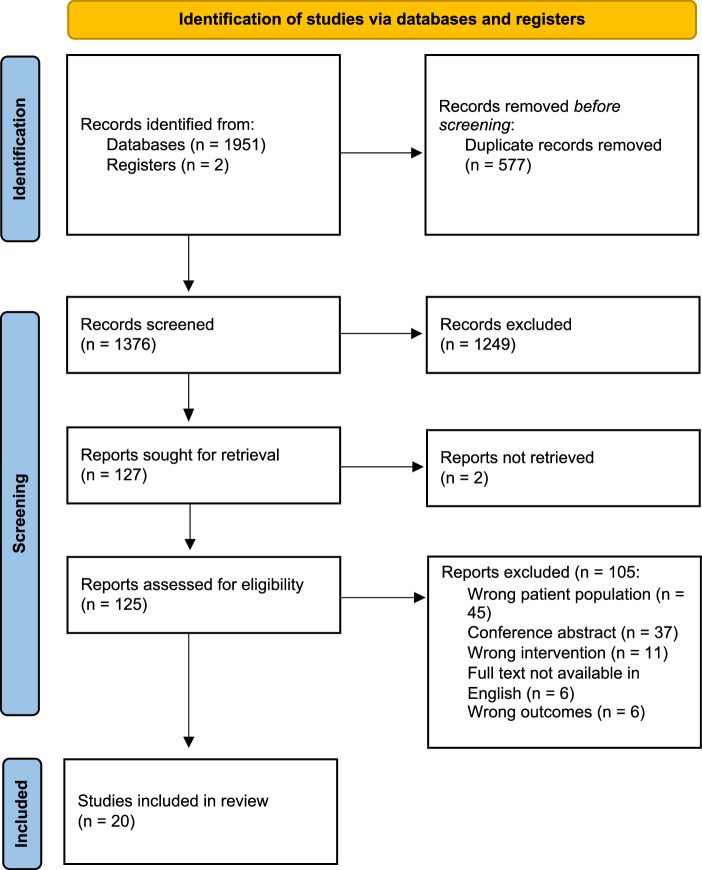
The PRISMA flow diagram of studies included in the review.

Nearly 2000 articles were screened, and 20 studies were included in the final review^2,4,20–38^ (Fig. 1). A summary of all the baseline characteristics is available in Supplementary Table 2, Supplemental Digital Content 2, http://links.lww.com/SLA/F494. The studies were published between 2010 and 2024 in the United States, France, Switzerland, the Netherlands, Denmark, Norway, the United Kingdom, Turkey, and China. Four of the studies were RCTs,^4,24,27,38^ 5 were prospective cohort studies,^20,32,33,36,37^ 10 were retrospective cohort studies,^21–23,25,26,28–31,34,35^ with 1 case-controlled study.^35^ Nineteen studies utilized lytic therapies including both pharmacomechanical catheter-directed thrombolysis and CDT alone,^4,20–31,33–37^ while 1 study used purely mechanical thrombectomy.^32^ The majority of included studies were cohorts of patients with purely IF-DVT, and 2 studies included isolated femoral DVT as well.^2,37^

Intravascular ultrasound was used in 5 studies^20,25,28,32,36^ and anticoagulation was administered universally in all studies; adjunctive antiplatelets were also used in 3 studies.^20,24,34^ Compression therapies were administered in all but 3 studies.^33,34,36^ Symptom duration ranged from an average of 4.1 days to 9.1 days, with almost all patients treated within 28 days. In the CLOUT registry, 11.6% patients reported symptoms of >4 weeks duration.^32^ In 7 studies, BMI ranged from an average of 27.6 to 31.2.^20,21,24,27,28,32,35^ The VTE provocation factors reported by studies included malignancy, hypercoagulability, recent surgery, previous VTE, immobility, smoking, obesity, trauma, postpartum, and hormone therapies. Overall, there were 2683 participants, of whom 51.3% were female, with an average age of 31 to 65 years. A median of 51.8% (IQR: 12.2–74.0%) of patients received venoplasty in the 9 studies in which it was reported,^2,4,22–25,29,30,33^ and a median of 46.3% (IQR: 7.9–55.9%) received a stent as reported in 17 studies.^2,4,20–26,28–31,33,35–37^

### Procedural Characteristics

The devices used in the studies included Angiojet (Boston Scientific),^4,20,22,24,25,28,35,36^ EkoSonic (EKOS Corporation),^20,27,30,36^ Cleaner (Argon Medical),^29^ Mantis (Invamed),^21^ PTD-Arrow-Trerotola (Teleflex, Inc.),^34^ Trellis (Covidien),^4,20,24^ Uni-Fuse (AngioDynamics),^2,23,26,30,31,35^ and ClotTriever (Inari Medical).^32^

A number of different definitions for successful lysis were used in the studies. The most common definition was lysis grade, with <50% patency classed as grade I (failure) thrombolysis, grade II lysis as 50% to 95% patency following intervention, and grade III as >95% patency, used in 5 studies.^22,29–31,35^ Other definitions for lysis success were as follows: >50% (success) or <50% (failure) patency following intervention or recurrence of DVT within 30 days^20,23^; >90% patency following intervention (success)^28,33^; antegrade flow and maximal luminal stenosis of 30% assessed on final procedural venography^36^; and removal of thrombus, restoration of inline flow, and venous patency as much as possible.^4,34,37^ Two studies reported Marder score change after treatment.^21,24^ Because of the heterogeneity in the application of these definitions and categorical reporting of data, it was not possible to perform meta-analysis. A summary of outcomes for each study is presented in Supplementary Table 3, Supplemental Digital Content 2, http://links.lww.com/SLA/F494, while Table 1 contains the pooled outcomes for each treatment strategy (lytic therapies, mechanical thrombectomy, and anticoagulation).

**TABLE 1:** The Pooled Meta-analysis Outcomes by Intervention Strategy

	Pharmacomechanical thrombectomy	Mechanical thrombectomy	Anticoagulation alone
PTS at maximal follow-up	24.5% (95% CI: 19.5%–30.3%) *I* ^2^=88% N=2212	18.8% N=213	46.0% (95% CI: 40.6%–51.5%) *I* ^2^=90% N=351
Moderate-severe PTS at maximal follow-up	11.7% (95% CI: 9.7%–13.9%) *I* ^2^=82% N=1346	8.0% N=213	17.8% (95% CI: 14.1%–22.2%) *I* ^2^=24% N=351
Major bleeding	3.1% (95% CI: 2.2%–4.2%) *I* ^2^=3% N=1856	0.0% N=213	0.8% (95% CI: 0.1–5.1) N=351
Ulceration	4.2% (95% CI: 2.2%–7.8%) *I* ^2^=58% N=999	Not reported	9.0% (95% CI: 5.2%–15.2%) N=222
Peri-op re-thrombosis	5.4% (95% CI: 3.0%–9.8%) *I* ^2^=88% N=1030	6.0% N=184	NA
DVT recurrence during follow-up	9.2% (95% CI: 6.5%–12.9%) *I* ^2^=71% N=1269	Not reported	15.6% (95% CI: 11.8%–20.4%) *I* ^2^=17% N=280
Treatment-related mortality	Multiple deaths across studies, with only 1 directly attributed to treatment: massive PE secondary to thrombus migration following incomplete balloon occlusion of the IVC	One death following the entanglement of the ClotTriever device with another medical device, resulting in pulmonary embolism	Multiple deaths across studies, but none directly attributed to treatment

### PTS Rates

#### RCTs of Early Thrombus Removal Versus BMT

**FIGURE 2:**
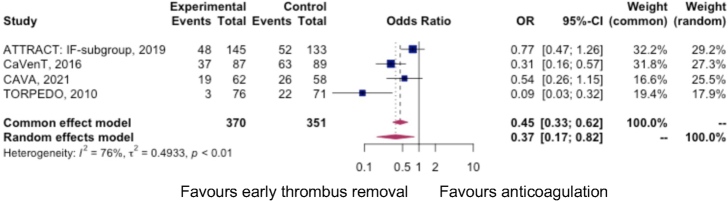
Forest plot summarizing the instance of PTS in randomized controlled trial arms.

In the 4 RCTs, there were 370 patients who received interventional therapy, all with lytic therapies, followed by anticoagulation, with 107 instances of PTS as defined by Villalta score 5 or more.^2,4,24,27^ There were 351 patients who received anticoagulation alone, of whom 163 patients developed PTS. The odds of developing any grade of PTS were 0.45 (95% CI: 0.33–0.62, *I* ^2^=76%) with early thrombus removal as compared with treatment with anticoagulation alone, with a number needed to treat (NNT) of 6 (95% CI: 5–10). Figure 2 demonstrates a forest plot summary of the PTS outcomes in each trial.

As PTS is known to evolve over time, the rate of PTS with early thrombus removal at all time points was assessed in all groups. In the mechanical thrombectomy cohort, the rate of PTS was 18.8% (1 study, 40 events, and 213 participants) at 12 months of follow-up.^32^ Taking the longest possible follow-up, the overall rate of PTS with lytic therapies was 24.5% (95% CI: 19.5%–30.3%) (19 studies, 561 events, and 2212 participants, *I* ^2^=88%).^2,4,20–31,33–37^ In the control group, the rate of PTS was 46.0% (95% CI: 40.6%–51.5%) (4 studies, 163 events, and 351 participants, *I* ^2^=90%). Due to the high degree of heterogeneity, meta-regression was undertaken. Length of follow-up at the time of PTS assessment accounted for 31% and 99% of the total heterogeneity in the lytic therapies and control groups, respectively (*P*<0.01).

**FIGURE 3:**
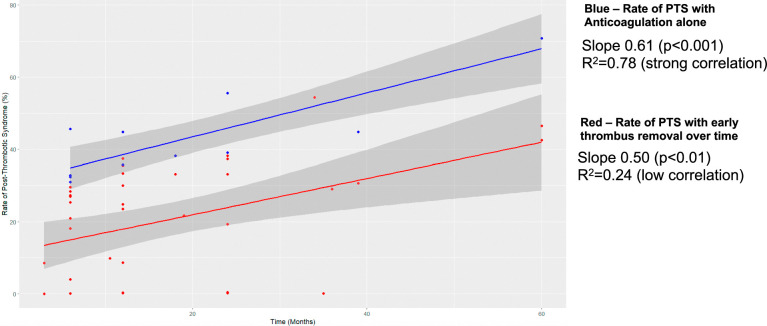
PTS rates at all reported time points with early thrombus removal versus anticoagulation.

In light of this finding, a linear regression analysis was undertaken by including the reported rate of PTS at all time points across the studies. In the intervention group with early thrombus removal, the intercept was 11.9% (*P*<0.01) and the slope value was 0.50 (*P*<0.01) with an *R* ^2^ value of 0.24 (low correlation). In the anticoagulation-alone group, the intercept was 31.2% (*P*<0.0001) and the slope was 0.61 (*P*<0.001) with an *R* ^2^ value of 0.78 (strong correlation). Figure 3 demonstrates a graph of the rate of PTS development over time with early thrombus removal versus anticoagulation alone.

### Moderate-Severe PTS

#### RCTs of Early Thrombus Removal Versus BMT

**FIGURE 4:**
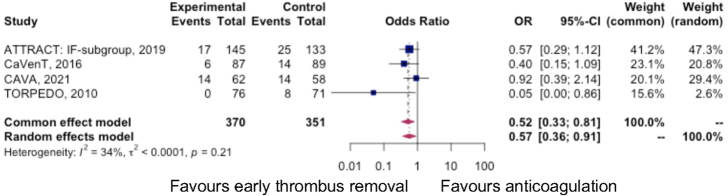
Forest plot summarizing the instance of moderate-severe PTS in randomized controlled trial arms.

Thirty-seven patients developed moderate-severe PTS (defined as Villalta score of 10 or more, or venous ulceration). The odds of developing moderate-severe PTS were 0.52 (95% CI: 0.33–0.81, *I* ^2^=0%) in the intervention arm, with an NNT of 14 (95% CI: 9–34). Figure 4 demonstrates a summary of the rates of moderate-severe PTS in each trial.

### PTS in All Studies

Considering all time points, the overall rate of moderate-severe PTS in the lytic therapy studies was 11.7% (95% CI: 9.7%–13.9%) (13 studies, 109 events, and 1346 participants, *I* ^2^=82%).^2,4,24,27–31,33–37^ The rate of moderate-severe PTS with mechanical thrombectomy was 8.0% (1 study, 17 events, and 213 participants) at 12 months of follow-up.^32^ In the anticoagulation-alone group, the rate of moderate-severe PTS was 17.8% (95% CI: 14.1%–22.2%) (4 studies, 61 events, and 351 participants, *I* ^2^=24%).^2,4,24,27^ Meta-regression demonstrated that duration of follow-up did not influence the likelihood of developing moderate-severe PTS in both the intervention and control (*R* ^2^=0.0%, *P*>0.05). A linear regression analysis was performed using the rate of PTS to evaluate the development of moderate-severe PTS across all time points from the included studies, which did not demonstrate any significance.

### Major Bleeding

#### RCTs of Early Thrombus Removal Versus BMT

**FIGURE 5:**
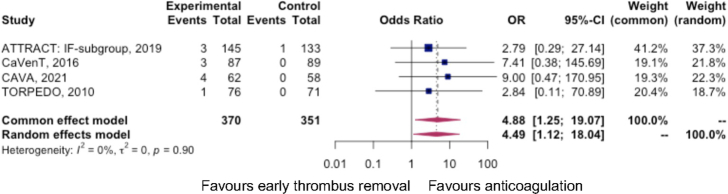
Forest plot summarizing the major bleeding events in randomized controlled trial arms.

Of the 370 participants receiving intervention in the RCT arms, there were 11 major bleeds, defined by the International Society on Thrombosis and Haemostasis as fatal bleeding, and/or symptomatic bleeding in a critical area or organ, and/or bleeding causing a fall in hemoglobin levels of 1.24 mmol/L (20 g/L) or greater, or leading to a transfusion of 2 units or more of blood. In the anticoagulation-alone arm, there was one major bleed among the 351 participants. The odds of major bleeding were 4.9 (95% CI: 1.3–19.1, *I* ^2^=0%). The number needed to harm (NNH) was 33 (95% CI: 20–100).^2,4,24,27^ Figure 5 demonstrates a forest plot of the pooled results.

Given the significantly increased odds of major bleeding in the intervention arm, each of the bleeding events was considered in turn. In the TORPEDO trial, the one major bleeding event was due to a drop in the hemoglobin of 20 g/L that did not result in transfusion.^4^ In the ATTRACT IF-DVT subgroup, there were 3 major bleeds in the intervention group and 1 in the control, with no fatal or intracranial bleeds.^24^ Though the exact nature of the events was not specified for the subgroups, in the original ATTRACT trial, there were 6 major bleeding events in the intervention group, of which 2 were gastrointestinal, 2 were retroperitoneal bleeds that required embolization and blood transfusion, and 2 were at the catheter access site. The major bleed in the control group was a gastrointestinal bleed.^5^ In the CaVenT trial, the major bleeding events were an abdominal wall hematoma necessitating blood transfusion, compartment syndrome of the calf needing fasciotomies, and 1 inguinal puncture site hematoma.^2^ In the CAVA trial, there were 4 major bleeds all in the intervention arm, 2 of which were puncture site bleeds resulting in >20 g/L drop in hemoglobin, 1 was a 40 g/L drop in hemoglobin due to a retroperitoneal bleed, and 1 was caused by the rupture of the femoral vein during sheath placement necessitating embolization.^3^

#### The Pooled Rate of Major Bleeding for Early Thrombus Removal

The rate of major bleeding was reported in 17 of the studies of lytic therapy. The overall rate of major bleeding was 3.1% (95% CI: 2.2%–4.2%) (17 studies, 36 events, and 1856 participants, *I* ^2^=3%).^2,4,20–25,27–31,33–36^ The rate of major bleeding in the mechanical thrombectomy study was 0%^32^ and for the anticoagulation-alone group was 0.8% (95% CI: 0.1–5.1) (4 studies, 1 event, and 351 participants).^2,4,24,27^

### Risk-Benefit Ratio

To reconcile the primary efficacy and primary safety outcomes, a risk-benefit ratio of the NNT:NNH was calculated. Supplemental Figure 1, Supplemental Digital Content 2, http://links.lww.com/SLA/F494, demonstrates a diagrammatic representation of the main risks and benefits of early thrombus removal, according to the evidence from RCTs and is designed to be used as a patient support tool. Supplemental Figure 1A, Supplemental Digital Content 2, http://links.lww.com/SLA/F494, demonstrates for every 100 patients treated, the number that will avoid PTS (16 patients) and the number that will also have a major bleed (3 patients) with lytic therapies (risk-benefit ratio 1:5). Supplemental Figure 1B, Supplemental Digital Content 2, http://links.lww.com/SLA/F494, demonstrates for every 100 patients treated, the number of patients that will avoid moderate-severe PTS (6 patients) and the number of patients that will develop a major bleed (3 patients) (risk-benefit ratio 1:2).

### Ulceration

In the lytic group, the rate of venous ulceration was 4.2% (95% CI: 2.2%–7.8%) (8 studies, 27 events, and 999 participants, *I* ^2^=58%).^2,21,24,31,33,35–37^ Ulceration was not reported in the mechanical thrombectomy group.^32^ Only 2 studies reported ulceration in the anticoagulation-alone group; the rate of ulceration was 9.0% (95% CI: 5.2%–15.2%) (2 studies, 12 events, and 222 participants).^2,24^ Table 1 contains all the secondary outcomes recorded in each study.

### Re-thrombosis and In-stent Stenosis

In the studies of lytic therapies, the rate of perioperative re-thrombosis/ re-occlusion was 5.4% (95% CI: 3.0%–9.8%) (9 studies, 75 events, and 1030 participants, *I* ^2^=88%).^20,23,24,30,31,34–37^ This was similar to the rate of perioperative re-thrombosis in the mechanical thrombectomy group at 6.0%^32^ (Table 1).

### DVT Recurrence During Follow-up

The rate of recurrence of DVT in the studies of lytic therapies was 9.2% (95% CI: 6.5%–12.9%) (12 studies, 136 events, and 1269 participants, *I* ^2^=71%).^2,20–24,27,29–31,36,37^ In the anticoagulation group, the rate of DVT recurrence was similar at 15.6% (95% CI: 11.8%–20.4%) (3 studies, 43 events, and 280 participants, *I* ^2^=17%)^2,24,27^ (Table 1). The mechanical thrombectomy data received did not report the rate of DVT recurrence after the immediate perioperative period.^32^

### Treatment-related Mortality

Mortality was reported in 11 of the lytic therapy studies.^2,4,20,21,24,27,31,33–36^ While there were deaths across the studies, only 1 death was attributed to the treatment received.^34^ During lysis, incomplete balloon occlusion of the inferior vena cava led to thrombus migration and massive pulmonary embolism. The patient was placed on extra-corporeal membrane oxygenation but died shortly afterward.^34^ There were no treatment-related deaths in the anticoagulation-alone group.^2,4,24,27^ In the mechanical thrombectomy registry, there was 1 treatment-related mortality that occurred through the concurrent use of the ClotTriever catheter with another device, resulting in the subsequent entanglement of the devices and the embolization of inferior vena cava thrombus to the lung.^32^

### Risk of Bias

Supplemental Tables 4 and 5, Supplemental Digital Content 2, http://links.lww.com/SLA/F494, display the risk of bias assessment for the randomized and nonrandomized studies of interventions, respectively. Four RCTs were evaluated using the Cochrane RoB^2,4,24,27^ (Supplemental Table 4, Supplemental Digital Content 2, http://links.lww.com/SLA/F494). All the RCTs demonstrated incomplete outcome data; however, the data were handled with a modified intention-to-treat analysis and therefore regarded as being at low risk of attrition bias, except for the TORPEDO trial, which was judged as having an unclear risk of bias across all domains. All 3 other RCTs were at low risk of selection bias, allocation was adequately concealed, and all assessors were blinded to patients’ allocation. Due to the nature of the intervention, it was challenging to blind the participants, but the knowledge of the intervention by the participants was deemed to have a low risk of influencing outcomes.

The rest of the studies were nonrandomized, and appraisal using the ROBINS-I tool revealed serious or critical risk of bias in all the observational studies^20–23,25,26,28–37^ (Supplemental Table 5, Supplemental Digital Content 2, http://links.lww.com/SLA/F494). Chiefly, this was due to selection bias and confounding; methods of recruitment were vaguely described, and confounding factors were not adequately controlled for, with the majority of studies being retrospectively conducted.

### GRADE Assessment

The quality of evidence from the RCTs included in the head-to-head meta-analysis was graded as high, with satisfactory directness, precision, consistency, and low risk of bias. The quality of evidence for the pooled rates of PTS, severe PTS, recurrent DVT, major bleeding, and mortality was downgraded to low given the serious risk of bias from some individual observational studies.

## DISCUSSION

This systematic review and meta-analysis has demonstrated the efficacy of early thrombus removal technologies for patients with IF-DVT, which significantly reduces both the PTS and moderate-severe PTS. While data from individual studies or trials have at times failed to demonstrate overall efficacy, the pooling of the outcomes and linear regression analysis has demonstrated that early thrombus removal confers a significant reduction in the rate of PTS across all time points, as well as the overall rate of moderate-severe PTS in the randomized trials. Overall, similar outcomes in both randomized and nonrandomized trials have permitted estimation of the rates of treatment benefits and treatment-related adverse events with reasonable precision.

Using meta-regression, a predictive model demonstrating the proportion of patients developing PTS over time was fitted to the data. The correlation is strong within the anticoagulation group, and the intercept of 31.2% indicates that without early thrombus removal, 31.2% of patients would be expected to develop PTS, while with intervention, that proportion of patients is reduced to 11.9%. Moreover, in the early thrombus removal group, the change in PTS rates over time is very weakly correlated, indicating that early thrombus removal may alter the likely disease course over time and prevent long-term progression to PTS. A mechanistic explanation for the finding could be that with early thrombus removal, there is a reduction in ongoing inflammation that limits further fibrosis that leads to PTS,^39^ hence rates remain more static over the follow-up, but this theory requires further investigation. To this end, it will be fascinating to see the outcomes of the DEXTERITY-AFP trial—using perivenous dexamethasone injection around the deep veins after removal of acute femoropopliteal DVT.^40^

Lytic therapies are not without risk of major bleeding, with a rate of >3% in the included studies and NNH of 33. The risk-benefit ratio is a crude measure that does not account for the importance and consequences of the outcome measures to individual patients; it has been demonstrated that for every 5 patients who avoid PTS, there is 1 major bleed, and for every 2 patients who avoid moderate-severe PTS, there is 1 major bleed. Though not leading to any treatment-related mortalities in the included studies, the major bleeds seen were at sites of potentially significant hemorrhage requiring transfusion and embolization, including gastrointestinal and retroperitoneal. Major bleeding remains a significant cause of patient concern and is an important patient-centered outcome measure.^41^ Major bleeding also increases length of stay, treatment costs, re-admission rates, and subsequent morbidity, all of which should be accounted for within the risk-benefit.^42–44^

This highlights the potential benefits of purely mechanical thrombectomy. The only lytic-free mechanical device included in this study was the ClotTriever catheter (Inari Medical). Mechanical thrombectomy has evolved as a treatment option over recent years, with several different devices now available: Indigo (Penumbra Inc.) and Aspirex S (Straub Medical AG), with evidence collection ongoing (NCT05701917, NCT03116750, and NCT05003843). In addition to reduced bleeding risk, mechanical thrombectomy has the benefit of extending the treatment window beyond 14 to 21 days and can be offered to patients at high risk of hemorrhage, such as those with recent trauma, surgery, or other indications for CDT. A retrospective review of 96 patients reported short-term outcomes for patients treated with a mechanical thrombectomy device ClotTriever; thrombus removal of >75% was achieved in 97% of patients and normal venous flow was seen in 97% of patients who completed the 30-day follow-up.^45^ In the cohort, 40% of patients had contraindications to thrombolytic therapy, highlighting the number of patients who currently do not have access to any early thrombus removal therapies. Long-term data and PTS rates were not reported, and therefore, the study was not included in this review.^45^ High-quality data from randomized trials are lacking; however, early outcomes for purely mechanical thrombectomy as presented in this study are comparable to lytic therapies (Table 1), without the bleeding risk.

The strengths of this study include the meta-analysis of the 4 head-to-head RCTs of early thrombus removal in the IF-DVT cohort, which demonstrated a low risk of bias and similar results when accounting for study size and duration of follow-up. The evidence was graded as high quality, and though the meta-analysis has included observational studies of low quality generating low certainty evidence, the concordance of the results between the observation and randomized studies has permitted comparisons to be drawn between lytic therapies and mechanical thrombectomy and anticoagulation. The result is a compendium (Fig. 5) that clinicians may consult to assess the risk-benefit of lytic therapies versus anticoagulation, including major bleeding and PTS accurately, to inform patients of the frequency of complications as well as the true risks/benefits of treatment. Conducting a mixed-linear regression analysis from all studies of early thrombus removal to model how PTS develops over time is also a useful indicator for both patients and clinicians to understand their probable disease course and the potential impact of treatment.

A weakness of this review is that it was not possible to meta-regress for stenting, compression or venous patency following early thrombus removal. This is due to heterogeneity in reporting and the inability to determine whether individual patients who received these co-interventions were those in whom outcomes, such as PTS, were improved. Even still, the literature surrounding the issue is uncertain. Previous works have suggested that venous patency and valvular competence should be maintained to reduce the incidence of PTS,^46^ and the CaVenT trial showed that at 6 months follow-up, iliofemoral patency was associated with PTS at 24 months.^2^ In a post-hoc analysis of the CAVA trial, successful lysis, defined as >90% restored patency, was not associated with lower PTS rates but with improved quality of life and stenting.^3^ The Open Vein hypothesis remains contentious and further research into the mechanisms of PTS are still desperately required.^47,48^

An additional methodological weakness is that the cohort of patients in the CaVenT trial was not all IF-DVT; of the 189 patients included in the study, 77 (41%) had IF-DVT, whereas the remaining 59% had femoral DVT above the mid-thigh.^2^ Even still, the data have been included as both the trial may represent a true cohort of patients that would be treated in clinical practice, is one of the landmark randomized studies in the field, and the inclusion of a small number (n=103) of femoral DVT would be expected to diminish the treatment effect rather than overestimate. For this reason, Broholm et al^37^ was also included, despite a small minority of patients presenting with isolated femoral DVT only.^37^

IF-DVT remains a severe presentation of venous thromboembolic disease with a high morbidity in the form of PTS. As the first meta-analysis of an explicitly IF-DVT cohort, this review has demonstrated the efficacy of early thrombus removal technologies in reducing the risk of PTS, including moderate-severe PTS, but has also highlighted the significant risk of major bleeding with lytic therapies. Evidence for mechanical thrombectomy in IF-DVT is currently limited to a single published study and a prospective registry; however, the technology demonstrates significant promise with similar rates of PTS and moderate-severe PTS at 6 months, without the risk of major bleeding. It is apparent that, going forward, both technologies are likely to be useful in the optimal management of DVT, and RCTs comparing the efficacy of each technology, or the benefits of multiple concurrent technologies, are urgently needed to optimize the treatment of IF-DVT.

## Supplementary Material
