# Supplementary material for: A Systematic Review and Meta-analysis of the Efficacy and Safety of Lytic and Non-lytic Early Thrombus Removal Technologies for Iliofemoral Deep Vein Thrombosis
Source: Ann Surg. 2025 May 27;283(2):225–33. doi: 10.1097/SLA.0000000000006765 (PMC12783348; doi:10.1097/SLA.0000000000006765)

**Supplementary Table 1 – Search terms used in the systematic review.**

| **Search terms** | |
| --- | --- |
| (DVT* OR Deep Vein Thromb* OR Iliofemoral thromb* OR Iliac vein thromb* OR proximal thromb* OR iliac thromb* OR vein*adj4 thromb*) | |
| AND | |
| (Thrombectomy OR Thrombectom* OR pharmacomechanical thrombectom* OR pharmaco-mechanical thrombectom* OR catheter directed thromb* OR PCDT OR thrombolysis) | |
| AND | |
| (Post thrombotic syndrome OR Post-thrombotic syndrome OR Postthrombotic syndrome OR Postphlebitic syndrome OR Chronic venous insufficiency OR Chronic venous disease OR Venous Ulcer* OR Venous Claudication OR Venous Ulceration OR PTS) | |
| Total n (MEDLINE, EMBASE, Cochrane) | 1754 |

| **Study Label**  **Supplementary Table 2 – Baseline characteristics for included studies** | **Country** | **Study Design** | **Comparison** | **Device** | **IVUS** | **Antithrombotic therapy** | **Compression therapy details** | **Symptom Duration** | **Age** | **Female : Male** | **BMI** | **Risk factors** | **N** | **Funding** |
| --- | --- | --- | --- | --- | --- | --- | --- | --- | --- | --- | --- | --- | --- | --- |
| ATTRACT: IF-subgroup | USA | RCT | PMT vs BMT |  | Not stated | LMWH followed by warfarin or rivaroxaban as per published guidelines. Antiplatelets in n=30 in thrombolysis arm, n=26 in control arm | Knee-high 30 - 40mmHg GCS up to 6 months with stockings replaced every 6 months | No difference in outcome between patients with symptoms for 0-1 and 1-2 weeks | Median 52 (IQR 39-62) | 191 : 208 | 31 (IQR 27-37) | Previous VTE (n=93), major surgery (n=40), hospitalisation (n=46), immobiliusation (n=10), postpartum (n=8) | 145 | The ATTRACT Trial was supported by grants from the National Heart, Lung, and Blood Institute (NHLBI) of the National Institutes of Health (NIH) for the clinical coordinating center. Boston Scientific and Covidien (now Medtronic) provided supplemental funding. Study drug and funding were provided by Genentech. Compression stockings were donated by BSN Medical. These companies played no role in the study’s design, conduct, analysis, or reporting. |
| Avgerinos 2016 | USA | Prospective cohort study | CDT + PMT | AngioJet, EKOS, multi-sidehole catheter, Trellis | Used selectively | Direct oral anticoagulant or low molecular weight heparin as per ACCP guidance for duration. Aspirin if iliac vein stenting. | Graduated elastic compression stockings (20-30 mm Hg) | 64 patients had symptoms <14 days, 29 patients had symptoms >14 days | 52 (16-83) | 47 : 46 | 31.2 (18.6 - 59.8) | Malignancy n=14, hypercoagulability disorder n=33, recent surgery n=19, previous DVT n=28, immobility n=2, trauma n=2) | 93 | Nil |
| Broholm 2011 | Denmark | Prospective cohort study | CDT | multiple side-hole catheter with tip occlusion | Not stated | Oral anticoagulation for 1 year or more | Compression stockings class II (23-32 mm Hg) | Maximum 14 days | 31 (15-58) | 85 : 24 | Not stated | Thrombophilia (n=52), caval atresia (n=8) | 109 | Nil |
| Budak 2022 | Turkey | Retrospective cohort study | PMT | Mantis catheter; and Dovi aspiration device and Viper Catheter with multiple side holes (Invamed) | No | LMWH for 6 months then apixaban 5 mg twice daily for patients without malignancy for 6 months to complete 1 year of treatment | Thigh-high elastic compression stockings, providing 30 to 40 mm Hg of pressure for 6 months or more | 6 (1-14) days | 52.6 (22-79) | 84 : 146 | 29 (26-35) | Malignancy (n=58), recent surgery (n=100), immobilisation (n=52), smoking (n=86), obesity (n=19), hypercoagulabillity (n=34), trauma (n=24) | 230 | Nil |
| Cao 2020 | China | Retrospective cohort study | PMT | AngioJet | Not stated | LMWH til discharge then rivaroxaban 20mg OD for at least 6 months | 30-40mmHg compression socks for 6 months or more | <14 days | 56.2 (27-87) | 62 : 20 | Not stated | Malignancy (n=2), recent surgery (N=5), fracture (N=11), postpartum (N=9) | 82 | Not reported |
| CAVA | Netherlands | RCT | PMT vs BMT | EKOS | Not stated | Vitamin K antagonists, DOACs or LMWH according to international guidelines | Knee-high 30 - 40mmHg GCS during Waking hours every day for a minimum of 24 months | 6.0 (3-11) days | 49.0 (37.3-63.8) | 63 : 57 | 27.6 ±4.8 | Surgery (n=14), trauma (n=5), pregnancy or childbirth (n=12), hormone therapy (n=22), prev VTE (n=16), malignancy (n=2) | 120 | The CAVA trial was funded by a grant from ZonMw (The Netherlands Organisation for Health Research and Development, project number 171101001) and additional funding was provided by the board of the Maastricht University Medical Centre. The funders of this study had no role in the study design, data collection, data analysis, data interpretation, or writing of the report. |
| CaVenT | Norway | RCT | CDT vs BMT | Uni*Fuse Infusion Catheter | Not stated | Warfarin | Class II elastic compression stockings daily for 24 months | 4.4 ± 4.4 days in intervention arm, 6.8 ± 4.8 days in control arm | 51.5 ± 15.8 | 70 : 119 | Not stated | Malignancy (n=4), obesity (n=20), horme therapy (n=24), trauma (n=25), surgery (n=28), previous VTE (n=18), hypercoagulabillity (n=78) | 189 | Grants from Research Council of Norway, the South-Eastern Norway Health Authority, the University of Oslo, and Oslo University Hospital Ullevål |
| Che 2020 | China | Retrospective cohort study | CDT + stent vs CDT alone | Multiside hole catheter and a distal infusing wire (UniFuse; AngioDynamics) | Not stated | Stented patients received rivaroxaban 15mg BD for 20 days then OD for 6 months. Unstented patients received warfarin with a target INR 2-3. | Elastic compression stockings for left lower limbs | 7.2 ±4.7 | 50.8 ±11.5 | 45 : 83 | Not stated | Surgery or trauma (n=17), hypercoagulability (n=18), malignancy (n=8) | 83 | Not reported |
| CLOUT Registry: IF-subgroup | USA | Prospective cohort study | MT | ClotTriever System | Recommended pre- and post-procedure | Post thrombectomy anticoagulation regimen was unconstrained by the study protocol | Not stated | 88.4% of patients had symptoms <4 weeks | 61.9 (48.0–70.8) | In full cohort 252 : 247 | 30.2 (IQR 25.8–35.10 | In full cohort -  provoked DVT (n=206), history of DVT (n=124) | 213 | The CLOUT registry is sponsored by Inari Medical |
| Huang 2021 | China | Retrospective cohort study | PMT vs CDT | AnjoiJet and multiside hole catheter | In 19 patients | Warfarin or rivaroxaban for 3 months or more | Variable numbers of patients complied with compression stocking use up to 5 years post-op | 9.13 ±3.15 days for pharmacomechanical thrombectomy, 8.01±3.53 days for catheter directed thrombolysis | 56.03 ± 13.71 in pharmacomechanical thrombectomy groups vs 58.04 ± 17.34 in catheter directed thrombolysis group | 72 : 59 | Not stated | Malignancy (n=15), immobilisation (n=18), surgery (n=27), trauma (n=27), | 131 | Not reported |
| Huang 2023 | China | Prospective cohort study | CDT | 5F multi-side hole intravenous infusion catheter (Cook Medical) | Not stated | Warfarin for 3 months | Not stated | Maximum 14 days (mean 7.8 SD 1.9 days) | Mean 57.2 (SD 4.1) | In full cohort 61 : 42 | Mean 57.1 (SD 9.6) | In full cohort previous VTE (n=14), malignancy (n=6), May-Thurner syndrome (n=31), hip prosthesis (n=1), major surgery (n=4), trauma (n=9), childbirth (n=4) | 77 | This work was supported in part by the National Natural Science Foundation of China (81971607, 81729003), the Guangdong Medical Science and Technology Research Foundation (B2021376), Guangzhou Science and Technology Planning Project (202103000002), and Panyu Major Science Technology Planning Project (2020-Z04-002) |
| Kang 2024 | China | Retrospective case controlled study | PMT vs CDT | PMT = AngioJet catheter (6F Solent or 8F Zelante; Boston Scientific) CDT = Unifuse catheter (AngioDynamics) | Not stated | Enoxaparin until discharge, then rivaroxaban for 3 months or more | Venous stretch sock treatment for 6 months or more | ≤7 days (PMT = 4.1 +/- 1.1 days, CDT = 4.5 +/- 0.8 days) | Mean 64.93 (SD 14.79) | 56 : 39 | median 27 (IQR 24-34) in PMT group, median 28 (IQR 22-36) in CDT group | Surgery/trauma (n=31), May-Thurner syndrome (n=44), hypercoagulable state for tumour or immune disease (n=7) | 95 | Nil |
| Ming 2017 | China | Retrospective cohort study | CDT and stenting vs CDT alone | UniFuse Infusion Catheter (AngioDynamics) | Not stated | Warfarin for 6 months of more | Elastic compression stockings (class II, 30–40 mm Hg) were recommended as daily use for all the patients for 12 months or more | Not stated | Not stated | 133 : 114 | In patients without PTS 22.67_±_2.44, in patients with PTS 23.51_±_3.20 | Not stated | 247 | supported by Science and Technology Program of Nantong, China (MS22015050). |
| Pouncey 2020 | UK | Retrospective cohort study | PMT + CDT vs CDT alone | AngioJet | Yes | Oral anticoagulation with duration decision led by haematology | Graduated compression stocking provided in accordance with CHEST guidance | 5 (1–28) in PMT group, 4 (1–28) in CDT group | 51 (16–80) in PMT group, 44 (14–70) in CDT group | 74 : 65 | 28.2 (19–38.1) in PMT group, 26.5 (17.0–46.7) in CDT group | Smoking (n=28), trauma (n=9), malignancy (n=10), postpartum (n=7), thrombophilia (n=37) | 136 | Stephen Black has received consultancy fees from BSCI, Phillips-Volcano, Cook, Medtronic, Ekos-BTG, Optimed, Vesper, Veryan, Vetex, BD-Bard, and Bayer. |
| Rodoplu 2020 | Turkey | Retrospective cohort study | PMT | Cleaner | Not stated | LMWH, then Warfarin | Compression stockings at least for 3 months | 7.2 ± 5.6 days (range: 1–14 days) | 45.7 ± 18.9 years (range: 22-78 years) | Not stated | Not stated | Not stated | 54 | The author(s) received no financial support for the research, authorship, and/or publication of this article. |
| Swiss Venous Stent Registry | Switzerland | Prospective cohort study | PMT vs CDT vs CDT with bail out PMT | PMT = AngioJet ZelanteDVTTM catheter, CDT = EKOS catheter | Used selectively with the 83 patients undergoing EKOS | Vitamin K antagonists or direct oral anticoagulants (rivaroxaban, apixaban, or dabigatran) | Not stated | <14 days | mean 49 ± SD 20 | 93 : 66 | Not stated | Immobilisation (n=50), OCP/oestrogen therapy (n=41), previous VTE (n=35), chronic venous disease (n=24), major surgery (n=14), trauma (n=12), rheumatic disease (n=6), active cancer (n=6) | 159 | Not reported |
| Thony 2023 | France | Retrospective cohort study | PMT | Slow rotation with large tip (PTD-Arrow-Trerotola; Teleflex, Inc., Wayne, PA, USA) | Not stated | Oral anticoagulation (warfarin, acenocoumarol, apixaban, rivaroxaban) for 6 months, Aspirin (75-160mg/d) for 1 month | Not stated | 8.0 ± 5.8 days | 45 ± 20 (range 13-91) | In full cohort 164 : 113 | Not stated | In full cohort immobilisation (n=66), OCP (n=35), surgery (n=22), pregnancy/post-partum (n=23), medical disease - APS/bechet/other - (n=27), inherited thrombophilia (n=33), malignancy (n=14), May-Thurner syndrome (n=136), congenital atresia of IVC (n=28), post-thrombotic venous lesions (n=29), venous stenosis or compression (n=31) | 181 | Nil |
| Tichelaar 2016 | Norway | Retrospective cohort study | CDT | EKOS EkoSonic® Endovascular System with MACH 4e or Multiple side hole catheter (UNI-FUSE, AngioDynamics or Cragg-McNamara® Valved Infusion Catheter) | Not stated | Warfarin for one year or indefinitely if stented | Used but not specified in what number | In group 1 4.0 (1.0–7.0) days vs  3.0 (1.0–9.0) days in group 2 | 49.5 (34.0–62.3) in group 1 vs 34.0 (21.5–57.0) in group 2 | 29 : 57 | Not stated | Surgery (n=10), trauma (n=7), immobilisation (n=2), malignancy (n=10), puerperium (n=6), hormone use (n=19), obesity (n=24), thrombophilia (n=6), previous VTE (n=17) | 95 | Not reported |
| TORPEDO | USA | RCT | PMT vs BMT | AngioJet, Trellis | Not stated | Warfarin | thigh-high-graded compression stockings at 30–40 mm Hg for 6 months or more | 5 days | 61±11.3 in intervention group versus 61±10.2 control group | In full cohort 90 : 103 | Not stated | In full cohort, smoking (n=17), surgery or trauma (n=24), hormone therapy (n=11), malignancy (n=20) | 147 | Not reported |
| Wang 2018 | China | Retrospective cohort study | CDT | 4F infusion catheter (Uni*Fuse; AngioDynamics, Latham, NY) with multiple holes | No | Warfarin for 3 months or more | graduated elastic compression stockings (30-40 mm Hg) for 12 months or more | 5.76 days (range, 1-14 days) | 54.55 years (range, 21-70 years) | 69 : 56 | Not stated | Surgery (n=17), trauma (n=15), malignancy (n=13), immobillity (n=10), steroids (n=3), thrombophilia (n=7), smoking (n=60) | 97 | This study was funded by National Natural Science Foundation of China (Grant No. 81470583). |

**Table Legend**

ACCP – American College of Chest Physicians, APS – anti-phospholipid syndrome, BD – twice a day, BMT – best medical therapy, CDT – catheter-directed thrombolysis, DOAC – direct oral anticoagulant, DVT – deep vein thrombosis, GCS – graduated compression stockings, IQR – interquartile range, IVC – inferior vena cava, LMWH – low molecular weight heparin, MT – mechanical thrombectomy, OCP – oral contraceptive pill, OD – once a day, PMT – pharmacomechanical thrombectomy, PTS – post thrombotic syndrome, RCT – randomised controlled trial, VTE – venous thromboembolism

| **Study Label**  **Supplementary Table 3 – Recorded outcomes for included studies** | **Number of patients** | **Angioplasty** | **Stenting** | **Patency** | **Lysis efficacy** | **PTS** | **Moderate-Severe PTS** | **Ulceration** | **Major Bleeding** | **Peri-operative re-thrombosis** | **Recurrent DVT** | **Treatment related mortality** | **Mean follow up** |
| --- | --- | --- | --- | --- | --- | --- | --- | --- | --- | --- | --- | --- | --- |
| ATTRACT: IF-subgroup | 145 | 128 (88.3%) | 70 (48.3%) | Not stated | Mean thrombus removal 86%; Marder score change from a median of 11 (IQR 8-16) prelysis to 2 post lysis (IQR 0-4) | 48 (33.1%) vs 52 (39.1%) in anticoagulation alone cohort | 17 (11.7%) vs 25 (18.8%) in anticoagulation alone group | 9 (6.2%) vs 12 (9.0%) in anticoagulation alone group | 3 (2.1%) vs 1 (0.7%) in anticoagulation alone group | 11 (7.6%) | 26 (17.9%) vs 18 (13.5%) in anticoagulation alone group | 6 (4.1%) in both groups - not specified whether or not related to treatment, none within 10 days | 24 months |
| Avgerinos 2016 | 93 | Not reported | 52 (55.9%) | 1-, 2-, and 3-year primary venous limb patency rates of 82.7%, 77.9%, and 72.1%, respectively | Immediate treatment failure (<50% lysis or perioperative recurrence) was seen in 11 patients (12%). Seven of these had incomplete thrombolysis (≤50%), six of whom ended up with immediate recurrence. Four immediate recurrences occurred despite successful lysis (>50%). | 27 (29.0%) | Not reported | Not reported | 2 (2.2%) | 10 (10.8%) | 6 (6.5%) | One death after 25 days in a patient that received lysis for phlegmasia cerulea dolens; cause unclear | 36 months |
| Broholm 2011 | 109 | Not reported | 62 (56.9%) | At 6 years, the estimated percentage of patent veins without reflux was 87.5% | 3 patients had chronic occlusions not lysed, 2 had chronic changes not lysed, 3 had occlusion <1wk after CDT | 18 (16.5%) at >1 year follow-up (median follow-up 71 months) | 5 (4.6%) | 1 (0.9%) | Not reported | 3 (2.8%) | 3 (2.8%) | Not reported | Median follow-up was 71 months (range 15-124 months) |
| Budak 2022 | 230 | Not reported | 0 (0%) | Primary patency rate of 94.0%, 87.2%, and 81.7% at 1, 6, and 12 months | Marder score change from 12.65 to 2.4; Anatomic success was achieved in 95.2% of the patients, with PMT failure in 11 patients | 57 (24.8%) | Not reported | 8 (3.5%) | 0 (0.0%) | Not reported | 43 (18.7%) | None at 30 days | 12 months |
| Cao 2020 | 82 | 45 (54.9%) | 3 (3.7%) | The patency rate in patients undergoing stent implantation was 66.6% and that in patients undergoing PTA was 93.3% at follow up | Grade II lysis in 27/82 (32.9%) and Grade III lysis in 55/82 (67.1%) | 8 (8.8%) | Not reported | Not reported | 0 (0.0%) | Not reported | 5 (6.1%) | Not reported | 6–15 months (average follow-up duration: 10.5 ± 4.3 months) |
| CAVA | 120 | Not reported | Not reported | Not reported | NA | 19 (30.7%) vs 26 (44.8%) in anticoagulation alone cohort | 14 (22.6%) vs 14 (24.1%) in anticoagulation alone group | Not reported | 4 (6.5%) vs 0 (0.0%) in anticoagulation alone group | Not reported | 5 (8.1%) vs 7 (12.1%) in anticoagulation alone group | 0 (0.0%) vs 0 (0.0%) in anticoagulation alone group | Median follow up of 39.0 months (IQR, 23.3–63.8) |
| CaVenT | 189 | 23 (12.2%) | 15 (7.9%) | Not stated | Iliofemoral patency at 6 months 65.9% vs 47.4% in the intervention | 37 (42.5%) vs 63 (70.8%) in anticoagulation alone cohort | 6 (6.9%) vs 14 (15.7%) in anticoagulation alone group | 0 (0.0%) vs 0 (0.0%) in anticoagulation alone group | 3 (3.4%) vs 0 (0.0%) in anticoagulation alone group | Not reported | 10 (11.5%) vs 18 (20.2%) in anticoagulation alone group | 0 (0.0%) vs 0 (0.0%) in anticoagulation alone group | 60 months |
| Che 2020 | 83 | 43 (51.8%) | 43 (51.8%) | Primary patency at 6 months was 83.1% and secondary patency was 87% | All patients successfully underwent surgery | 15 (18.1%) | Not reported | Not reported | 2 (2.4%) | 3 (3.6%) | 10 (12.0%) | Not reported | 6 months |
| CLOUT Registry: IF-subgroup | 213 | 379 (72.7%) of total cohort of 499 patients | 231 (44.3%) of total cohort of 499 patients | 94% of IF patients had patency at 1 year | N/A | At the 1-year visit, 81.2% (n = 173) of limbs affected by IF DVT were free from PTS | 17 (8.0%) | Not reported | 0 (0.%) | 11 out of 184 patients (6.0%) | Not reported | One death following the entanglement of the ClotTriever device with another medical device resulting in pulmonary embolism | 12 months |
| Huang 2021 | 131 | 97 (74.0%) | 54 (41.2%) | Total patency at 5 years 45/83 (52%) | Not reported | 61 (46.6%) | Not reported | Not reported | 0 (0.0%) | Not reported | Not reported | Not reported | 60 months |
| Huang 2023 | 77 | 0 (0%) | 0 (0%) | Not reported | Average thrombolysis ratio after treatment = 78.1 SD 14.4 | The overall rate of PTS at 6, 12, and 24 months was 10.4%, 14.3%, and 22.1% | 0 (0%) | 0 (0%) | 2 (2.6%) | Not reported | Not reported | 0 (0%) | 24 months |
| Kang 2024 | 95 | In both groups, angioplasty of the stenosis lesion before catheter placement to improve efficiency of thrombolysis for some patients with severe May-Thurner syndrome – patient number not specified | 42 (44.2%) | Overall patency of 93.7% (89 patients) | 6 patients had grade I lysis (<50% vessel patency) | The incidence of PTS in the CDT-alone group and PCDT plus CDT group at 24 months after surgery was 47.0% and 27.7%, respectively | 23 (24.2%) | 8 (8.4%) | 4 (4.2%) | 2 (2.1%) | Not reported | 0 (0%) | 28 ± 4.2 months |
| Ming 2017 | 247 | Not reported | 116 (47.0%) | Not reported | Not reported | 74 (30.0%) | Not reported | Not reported | Not reported | Not reported | Not reported | Not reported | 12 months |
| Pouncey 2020 | 136 | Not reported | 119 (87.5%) | Cumulative patency at 85.4% for PCDT vs. 80.3% for CDT. | Lytic success in 122 participants | 32 (23.5%) | 9 (6.6%) | Not reported | 3 (2.2%) | Not reported | Not reported | Not reported | 12 months |
| Rodoplu 2020 | 54 | 7/82 (8.6%) of the original participants required balloon angioplasty | 0 (0%) in the acute phase | Not reported | Grade II lysis in 81/82 (98.7%); Grade III lysis in 75/82 (91.6%) | 18 (33.3%) | 9 (16.7%) | Not reported | 0 (0.0%) | Not reported | 1/82 (1/2%) of the original patients developed recurrent VTE requiring stenting | Not reported | 12 months |
| Swiss Venous Stent Registry | 159 | Not reported | 157 (98.7%) | The primary patency rate at 36 months was 85% (95%CI 79-91%): it was 92% (95%CI 83-100%) in the PMT alone group, 84% (95%CI 75-93%) in the CDT alone group, and 81% (95%CI 68-94%) in the CDT+PMT group | 5 patients developed early stent occlusion at 7 days | 12 (7.5%) | 2 (1.3%) | Not reported | 2 (1.3%) | 7 (4.4%) | 18 (11.3%) | 0 (0% | median follow-up was 35 (IQR 13-61) months |
| Thony 2023 | 181 | Not stated | Not stated | The patency rate at discharge (96.8%) was 87.7% after one intervention | A total (no thrombotic load) or subtotal (thrombotic load <10%) clearance of the venous thombosis was obtained at 96.1% of LET 3-4 locations, and 91% of LET 2 locations | 156 patients had a Villata score <4, giving a PTS rate (Villata >4) of 13.8% at 12 months | 7 (3.9%) | Not reported | 2 (1.1%) | 34 (18.8%) | Not reported | 1 (0.6%) occurring due to massive PE following incomplete seal of balloon occlusion of the IVC during PCDT | 12 months |
| Tichelaar 2016 | 95 | 88 (92.6%) | 44 (46.3%) | Complete patency 79 (CDT) vs. 76 % (USCDT). Stenosis in 3 patients, occlusion in 9 patients. |  | 37 (54.4%) | 10 (14.7%) | Not reported | 5 (7.4%) | 2 (2.9%) | 1 (1.5%) | Not reported | 89 months in one group and 34 months in another |
| TORPEDO | 147 | 68 (46.3%) | 27 (18.4%) | Not reported | Technical success was achieved in all patients irrespective of the used devices. | 3 (3.4%) vs 22 (31.0%) in anticoagulation alone cohort | 0 (0.0%) vs 8 (11.3%) in anticoagulation alone group | Not reported | 1 (1.3%) vs 0 (0.0%) in anticoagulation alone group | Not reported | Not reported | 0 (0.0%) vs 0 (0.0%) in anticoagulation alone group | 6 months |
| Wang 2018 | 97 | Not reported | 56 (57.7%) | The overall patency rate was 83.51% (81/97) during follow-up | Successful lysis (grade II and III lysis) was achieved in 92.37% (109/118) of patients | 21 (21.7%) | 7 (7.2%) | 1 (1.0%) | 3 (3.1%) | 3 (3.1%) | 8 (8.2%) | 0 (0.0%) | A total of 97 patients were followed up for a median of 19.39 ± 7.47 months (range, 6-32 months) |

**Table Legend**

BMT – best medical therapy, CDT – catheter-directed thrombolysis, CI – confidence interval, DVT – deep vein thrombosis, GCS – graduated compression stockings, IQR – interquartile range, MT – mechanical thrombectomy, NA – not applicable, OD – once a day, PMT – pharmacomechanical thrombectomy, PTS – post thrombotic syndrome, VTE – venous thromboembolism, USCDT – ultrasound guided catheter-directed thrombolysis

**Supplemental Table 4 –** **Risk of bias assessment of randomised controlled trials via the Cochrane risk-of-bias tool (RoB2).**

| **Author** | **Random sequence generation** | **Allocation concealment** | **Blinding (participants and personnel)** | **Blinding (outcome assessment)** | **Incomplete outcome data** | **Selective reporting** | **Overall** |
| --- | --- | --- | --- | --- | --- | --- | --- |
| ATTRACT | Low risk | Low risk | Low risk | Low risk | Low risk | Low risk | Low risk |
| CAVA | Low risk | Low risk | Low risk | Low risk | Low risk | Low risk | Low risk |
| CaVenT | Low risk | Low risk | Low risk | Low risk | Low risk | Low risk | Low risk |
| TORPEDO | Unclear | Unclear | Unclear | High risk | Low risk | Unclear | High risk |

**Supplemental Table 5 – Risk of bias assessment of non-randomised studies via the Risk of Bias in Non-Randomised Studies of Interventions (ROBINS-I) tool.**

| **Study** | **Bias due to confounding** | **Bias in selection of participants into the study** | **Bias in classification of interventions** | **Bias due to deviations from intended interventions** | **Bias due to missing data** | **Bias in measurement of outcomes** | **Bias in selection of the reported result** | **Overall Bias** |
| --- | --- | --- | --- | --- | --- | --- | --- | --- |
| Avgerinos 2016 | Serious risk | Serious risk | Low risk | Low risk | Critical risk | Serious risk | Serious risk | Critical risk |
| Broholm 2011 | Serious risk | Serious risk | Low risk | Low risk | Critical risk | Serious risk | Serious risk | Critical risk |
| Budak 2022 | Serious risk | Serious risk | Low risk | Moderate risk | Critical risk | Moderate risk | Serious risk | Critical risk |
| Cao 2020 | Serious risk | Critical risk | Low risk | Moderate risk | Critical risk | Serious risk | Critical risk | Critical risk |
| Che 2020 | Serious risk | Critical risk | Low risk | Moderate risk | Critical risk | Serious risk | Serious risk | Critical risk |
| CLOUT registry | Serious risk | Serious risk | Low risk | Low risk | Critical risk | Critical risk | Critical risk | Critical risk |
| Huang 2021 | Serious risk | Serious risk | Moderate risk | Serious risk | Critical risk | Serious risk | Serious risk | Critical risk |
| Huang 2023 | Serious risk | Serious risk | Moderate risk | Low risk | Critical risk | Serious risk | Serious risk | Critical risk |
| Kang 2024 | Serious risk | Critical risk | Critical risk | Moderate risk | Moderate risk | Serious risk | Serious risk | Critical risk |
| Ming 2017 | Serious risk | Critical risk | Moderate risk | Serious risk | Serious risk | Serious risk | Critical risk | Critical risk |
| Pouncey 2020 | Critical risk | Critical risk | Low risk | Low risk | Critical risk | Serious risk | Serious risk | Critical risk |
| Rodoplu 2021 | Serious risk | Critical risk | Low risk | Moderate risk | Critical risk | Serious risk | Serious risk | Critical risk |
| Swiss Venous Stent Registry | Serious risk | Serious risk | Low risk | Low risk | Moderate risk | Critical risk | Serious risk | Critical risk |
| Thony 2023 | Serious risk | Serious risk | Low risk | Moderate risk | Critical risk | Serious risk | Serious risk | Critical risk |
| Tichelaar 2016 | Serious risk | Serious risk | Moderate risk | Serious risk | Critical risk | Serious risk | Serious risk | Critical risk |
| Wang 2018 | Critical risk | Critical risk | Low risk | Low risk | Critical risk | Critical risk | Serious risk | Critical risk |

**Supplemental Figure 1 – The outcomes of 100 theoretical patients undergoing PCDT.** A visual representation of 100 theoretical patients undergoing LYTIC THERAPIES demonstrating the benefit (avoiding PTS) in green and the risk (major bleeding) in red. A) shows that 16 patients avoid PTS and three sustain a major non-fatal bleed per 100 patients treated. B) shows that, of the same 100 patients, six of the 16 patients will avoid moderate-severe PTS.


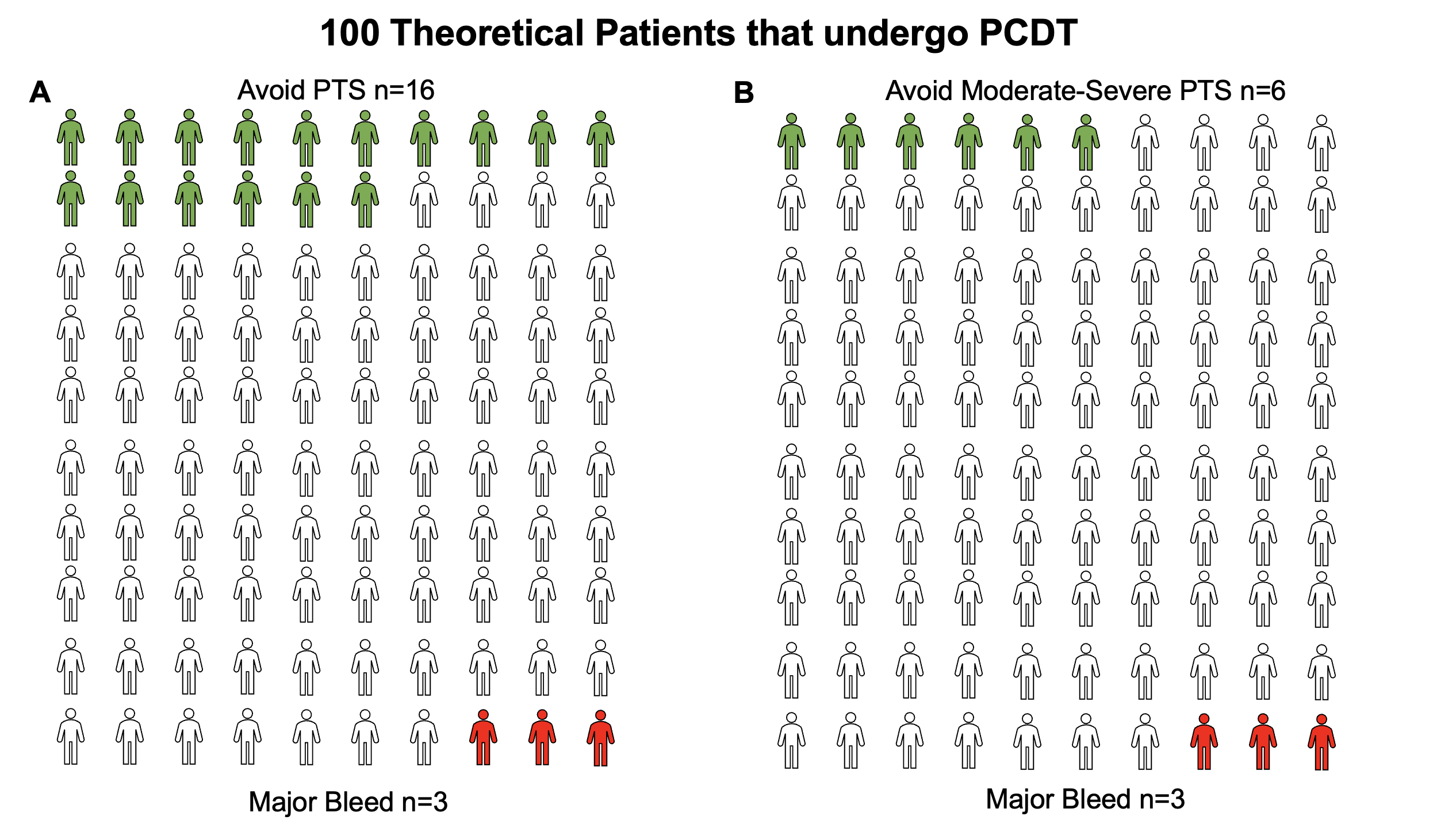

Supplement: Supplementary file 2 [file sla-283-225-s002.docx]
